# Multiple sclerosis as a model to investigate SARS‐CoV‐2 effect on brain atrophy

**DOI:** 10.1111/cns.14050

**Published:** 2022-12-07

**Authors:** Michael Rebsamen, Christoph Friedli, Piotr Radojewski, Lara Diem, Andrew Chan, Roland Wiest, Anke Salmen, Christian Rummel, Robert Hoepner

**Affiliations:** ^1^ Support Center for Advanced Neuroimaging (SCAN) University Institute of Diagnostic and Interventional Neuroradiology, Inselspital, Bern University Hospital and University of Bern Bern Switzerland; ^2^ Graduate School for Cellular and Biomedical Sciences University of Bern Bern Switzerland; ^3^ Department of Neurology Inselspital, Bern University Hospital and University of Bern Bern Switzerland; ^4^ Swiss Institute for Translational and Entrepreneurial Medicine, sitem‐insel Bern Switzerland

**Keywords:** brain atrophy, COVID, FreeSurfer, gray matter, volumetry

## Abstract

**Introduction:**

Data on structural brain changes after infection with SARS‐CoV‐2 is sparse. We postulate multiple sclerosis as a model to study the effects of SARS‐CoV‐2 on brain atrophy due to the unique availability of longitudinal imaging data in this patient group, enabling assessment of intraindividual brain atrophy rates.

**Methods:**

Global and regional cortical gray matter volumes were derived from structural MRIs using FreeSurfer. A linear model was fitted to the measures of the matching pre‐SARS‐CoV‐2 images with age as an explanatory variable. The residuals were used to determine whether the post‐SARS‐CoV‐2 volumes differed significantly from the baseline.

**Results:**

Fourteen RRMS patients with a total of 113 longitudinal magnetic resonance images were retrospectively analyzed. We found no acceleration of brain atrophy after infection with SARS‐CoV‐2 for global gray matter volume (*p* = 0.17). However, on the regional level, parahippocampal gyri showed a tendency toward volume reduction (*p* = 0.0076), suggesting accelerated atrophy during or after infection.

**Conclusions:**

Our results illustrate the opportunity of using longitudinal MRIs from existing MS registries to study brain changes associated with SARS‐CoV‐2 infections. We would like to address the global MS community with a call for action to use the available cohorts, reproduce the proposed analysis, and pool the results.

## Graphical abstract

Does infection with SARS‐CoV‐2 in MS patients lead to a change in brain atrophy? MS is an ideal model disease to study the potential effects of infection with SARS‐CoV‐2 on brain atrophy, as longitudinal clinical and imaging data are abundant. In this proof‐of‐principle study, we found no evidence of accelerated atrophy of global gray matter volume but accelerated atrophy in the parahippocampal gyri.

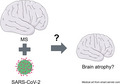

## 1 INTRODUCTION

Data on potential brain atrophy after severe acute respiratory syndrome coronavirus 2 (SARS‐CoV‐2) infection remains sparse and controversial. The until now largest study analyzing the United Kingdom (UK) Biobank data revealed changes in brain structure after infection with SARS‐CoV‐2 and regional atrophy patterns that differed from those of matched controls with no SARS‐CoV‐2 infection.^1^ In the group of 401 participants who tested positive for infection with SARS‐CoV‐2 between two magnetic resonance imaging (MRI) examinations, a greater reduction in gray matter thickness and tissue contrast in the orbitofrontal cortex and parahippocampal gyrus was observed. The authors underlined the need for additional follow‐up data to determine whether those findings are reversible or persistent in the long term. However, reliably deriving intraindividual brain atrophy rates requires multiple imaging examinations before the infection, which is not available with only two MRIs from the UK Biobank.

Multiple sclerosis (MS) is a chronic autoimmune demyelinating disease of the central nervous system with inflammatory and degenerative components.^2^ In individuals with MS, the risk of a severe course of SARS‐CoV‐2 infection is linked to age, certain immunotherapies, and patient characteristics such as ambulatory disability.^3^, ^4^

In our view, MS represents an ideal model for studying the potential effects of infection with SARS‐CoV‐2 on brain structure for several reasons. The main advantage of MS cohorts lies in the availability of imaging data with multiple scans at short follow‐up intervals both before and after infection with SARS‐CoV‐2 obtained with standardized imaging protocols. This allows adjustment for the intraindividual brain atrophy rate prior to SARS‐CoV‐2 infection. Thus, it is possible to determine whether the brain atrophy in a given individual accelerates either temporarily during the infection or persistently after the infection. Furthermore, brain atrophy in individuals with MS has been extensively studied and increased atrophy rates and differences in atrophy patterns in comparison to healthy individuals are well documented.^5^, ^6^ In particular, increased atrophy rates in spinal, striatal, pallidal, thalamic, and cortical areas as well as in the white matter and the corpus callosum have been described.^7^, ^8^ The orbitofrontal cortex and parahippocampal gyrus, which seem to be most affected by SARS‐CoV‐2 infection in the general population according to the UK Biobank study,^1^ are not known to be affected by MS. With this brief report, we propose to use MS registries worldwide to study SARS‐CoV‐2‐related brain atrophy specifically, by analyzing longitudinal MRI data on a patient level along the approach presented here.

## 2 METHODS

We identified 39 MS patients with an SARS‐CoV‐2 infection from our ongoing neuroimmunological registry (KEK BE 2017‐01369, Figure S1). Nine of those patients had incomplete follow‐up after SARS‐CoV‐2 infection (either missing clinical or MRI data) and were therefore excluded. To account for the possible influence of ongoing MS‐specific disease activity, we further excluded four patients with an unstable disease course. The remaining 26 patients fulfilled the criterion of “no evidence of disease activity” (NEDA‐3),^9^ demonstrating freedom from clinical relapses, no progression, and no new, enlarging, or enhancing lesions on MRI in the year before the SARS‐CoV‐2 infection. The patients were followed up according to the standard procedure at our MS center with at least one cerebral MRI examination on a 1.5 or 3T scanner per year and additional scans if new symptoms arose or when needed for pharmacovigilance. To increase comparability during follow‐up, patients are examined at the same field strength by default. MRI scans were acquired on Magnetom Vida 3T, Skyra 3T, Magnetom Avanto 1.5T, or Magnetom Aera 1.5T scanners (all Siemens Healthcare, Erlangen, Germany) with our standardized MS protocol.^10^ Atrophy was quantified on unenhanced, high‐resolution magnetization‐prepared rapid acquisition with gradient echo sequence (MPRAGE) images. MRI data were processed using the freely available software packages FreeSurfer (version 6.0.0) and DL+DiReCT to derive global and regional cortical gray matter (GM) volume and thickness.^11^, ^12^, ^13^

Patient‐specific atrophy rates prior to infection were calculated individually for every patient with at least three pre‐SARS‐CoV‐2 MRI scans that matched with at least one post‐SARS‐CoV‐2 scan with respect to field strength, repetition time, and inversion time (Figure S2), leading to the final cohort of 14 patients (Figure S1). A linear model was fitted to the global and regional GM volumes of the matching pre‐SARS‐CoV‐2 images using patient age as the explanatory variable. Standardized residuals were derived from matched pre‐ and post‐SARS‐CoV‐2 images and subjected to a two‐sided *t* test to determine whether the post‐SARS‐CoV‐2 residuals were significantly different from baseline residuals.

## 3 RESULTS

Fourteen patients with relapsing–remitting MS with a total of 113 eligible MR scans were finally included (Figure S3). The included patients had an average of 6.7 MRI scans (range 3–13) before and 1.4 scans (range 1–3) after SARS‐CoV‐2 infection. The mean MRI follow‐up interval after SARS‐CoV‐2 infection was 5.1 months (range 0.4–16 months). Patient characteristics are shown in Table 1. Four of the 14 patients were hospitalized due to SARS‐CoV‐2 infection, two of whom were treated in the intensive care unit. Four had been vaccinated with an mRNA vaccine before SARS‐CoV‐2 infection and four out of 14 patients received anti‐SARS‐CoV‐2 treatment (multiple treatments per patient possible; 2 x sotrovimab, 2 x remdesivir, 2 x dexamethasone, and 1 x convalescent serum). MS disease course remained stable after SARS‐CoV‐2 infection as no new or enlarging T2‐lesions or new gadolinium‐enhancing lesions were seen on the scans acquired postinfection. The expanded disability status scale (EDSS) remained stable for a mean of 10.4 months (range 0.7–20.1) after SARS‐CoV‐2 infection; mean EDSS before SARS‐CoV‐2 was 2.3 (range 0–6.0), whereas mean EDSS postinfection was 2.2 (range 0–6.0). No EDSS change was seen in nine patients, with 0.5 points improvement in three, and 0.5 points worsening in two patients.

**TABLE 1:** Patient characteristics

	Mean	Range	*N* (%)
Demographic and MS disease characteristics			
Age at infection with SARS‐CoV‐2 (years)	42	17–64	
Female sex			12/14 (85.7)
MS disease course: RRMS			14/14 (100)
Years since MS diagnosis	10.6	1.0–22.0	14/14 (100)
EDSS before SARS‐CoV‐2 infection	2.3	0–6.0	14/14 (100)
Months between the last EDSS assessment and the SARS‐CoV‐2 infection	5.3	0.2–11.0	
EDSS post‐SARS‐CoV‐2 infection	2.2	0–6.0	14/14 (100)
Months between the SARS‐CoV‐2 infection and the latest EDSS assessment	10.4	0.7–20.1	14/14 (100)
*Immunotherapy*			
Untreated			2/14 (14.29)
Fingolimod			3/14 (21.43)
Dimethyl fumarate			1/14 (7.1)
Rituximab			1/14 (7.1)
Natalizumab			1/14 (7.1)
Ocrelizumab			5/14 (35.71)
Ozanimod			1/14 (7.1)
SARS‐CoV‐2‐associated information			
*mRNA vaccination*			
none			10/14 (71.43)
2 doses			2/14 (14.29)
3 doses			2/14 (14.29)
Months between the last vaccine dose and the SARS‐CoV‐2 infection	4.2	1.3–7.5	4/4 (100.0)
*SARS‐CoV‐2 infection severity*			
Outpatient setting			10/14 (71.43)
Hospitalization			4/14 (28.57)
ICU			2/14 (14.29)
Death			0/14 (0.0)
*SARS‐CoV‐2 infection treatment*			
Remdesivir			2/14 (14.29)
Sotrovimab			2/14 (14.29)
Dexamethasone			2/14 (14.29)
Convalescent serum			1/14 (7.1)

Abbreviations: *EDSS*: expanded disability status scale; *ICU*: intensive care unit; *mRNA*: messenger ribonucleic acid; *N*: number of observations; *RRMS*: relapsing–remitting Multiple Sclerosis; *SARS‐CoV‐2*: severe acute respiratory syndrome coronavirus type 2.

**FIGURE 1:**
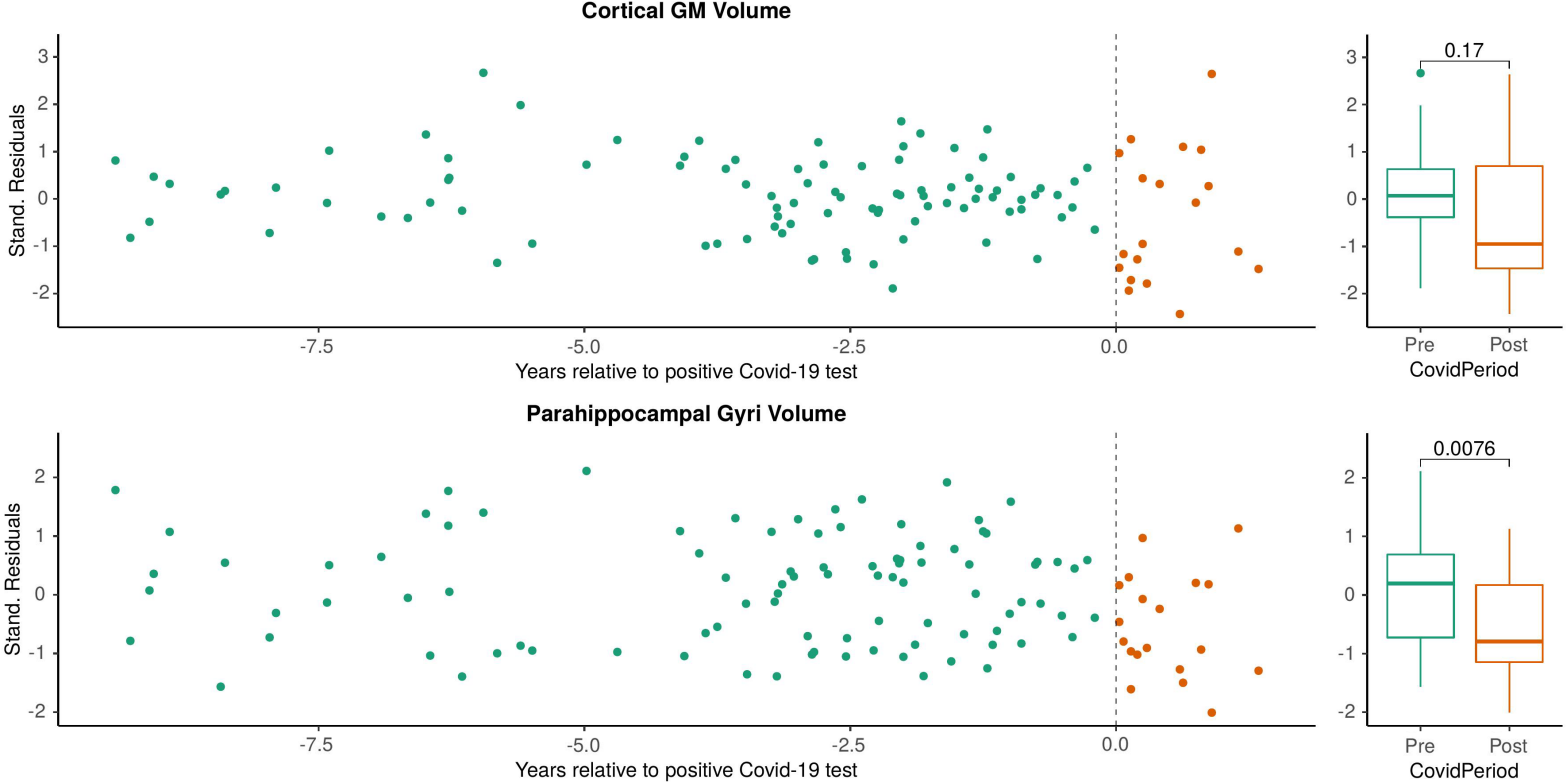
An increased atrophy rate of the parahippocampal gyri after SARS‐CoV‐2 infection using FreeSurfer for atrophy analysis. Longitudinal atrophy rates for individual patients are depicted in Figure S3. Analogous results from the DL+DiReCT software are presented in Figure S4. Abbreviations: COVID‐19: Corona Virus Disease 2019; GM: gray matter; SARS‐CoV‐2: severe acute respiratory syndrome coronavirus type 2; Stand.: standardized.

Using standardized residuals from the linear pre‐SARS‐CoV‐2 fits to adjust for the region‐ and person‐specific brain atrophy rate, the global GM volume showed no indication of accelerated brain atrophy during or after infection with SARS‐CoV‐2 (*p* = 0.17). However, the residuals of the parahippocampal gyri tended toward negative values (*p* = 0.0076), suggesting accelerated regional atrophy (Figure 1). These FreeSurfer‐generated results were confirmed with DL + DiReCT,^12^ an independent, deep learning‐based morphometry tool (Figure S4) and are in line with the findings from the UK Biobank study^1^ despite the younger age of our patients, their MS comorbidity and potentially different variants of SARS‐CoV‐2.

**FIGURE 2:**
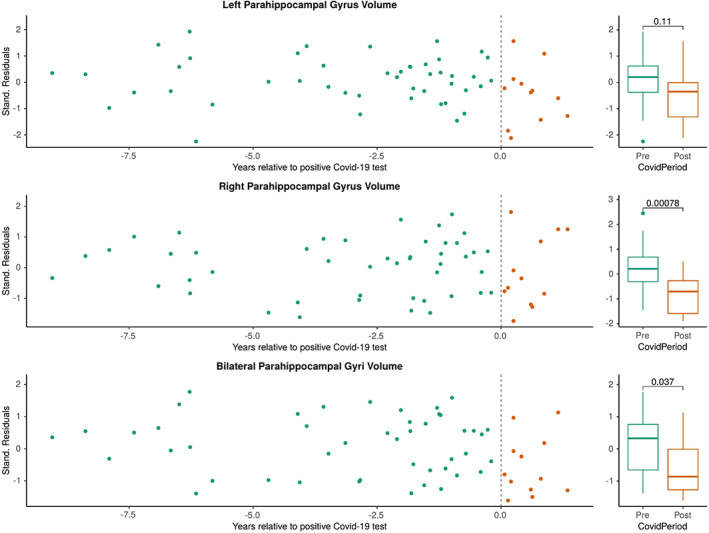
Subgroup analysis of patients who were unvaccinated and had received no anti‐SARS‐CoV‐2‐specific treatment. Abbreviations: COVID‐19: Corona Virus Disease 2019; SARS‐CoV‐2: severe acute respiratory syndrome coronavirus type 2; Stand.: standardized.

**FIGURE 3:**
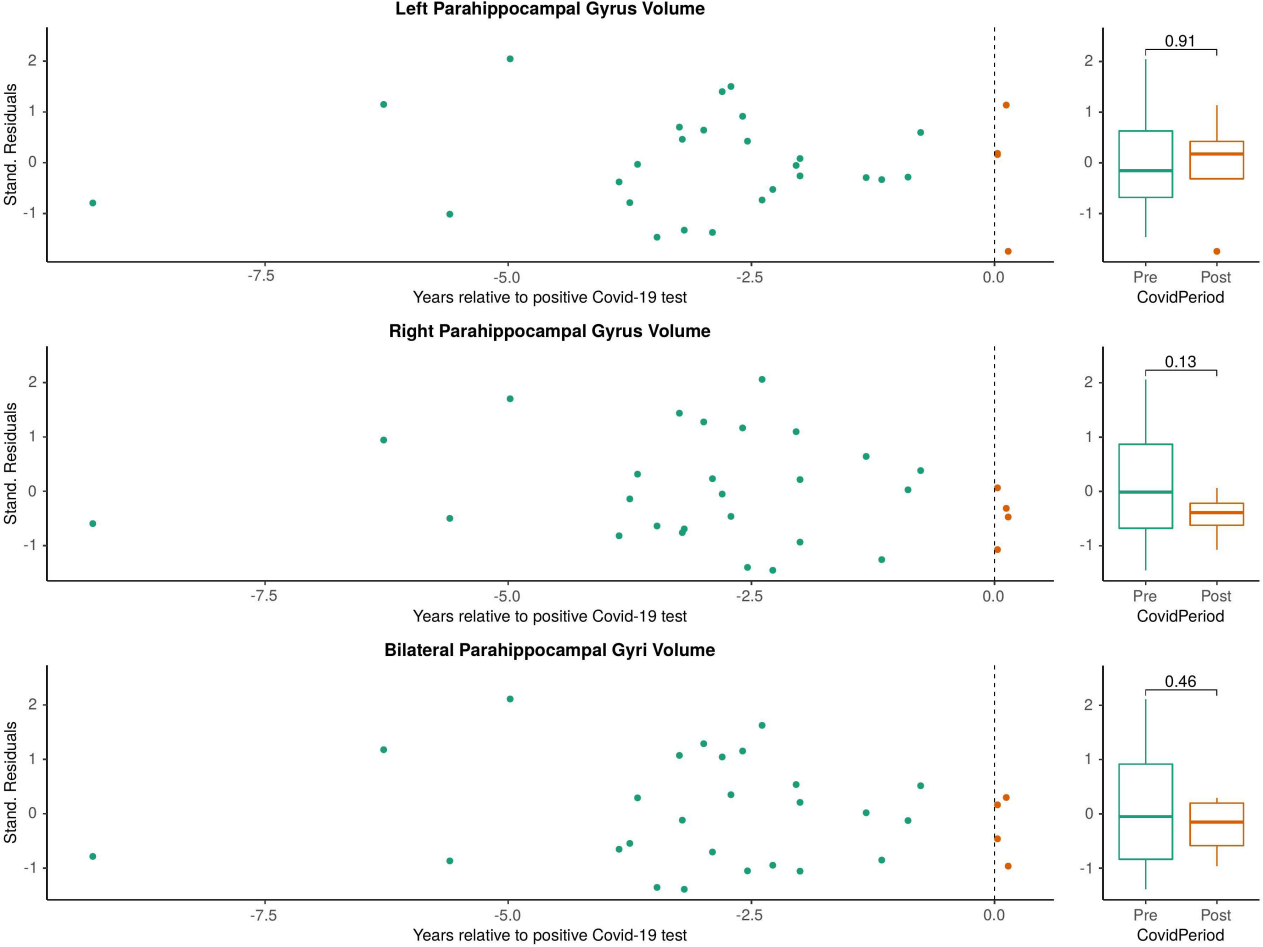
Subgroup analysis of patients who were vaccinated and/or had received anti‐SARS‐CoV‐2 specific treatment. Abbreviations: Covid‐19: Corona Virus Disease 2019; SARS‐CoV‐2: severe acute respiratory syndrome coronavirus type 2; Stand.: standardized.

Our cohort offered the potential for a preliminary investigation of the influence of antiviral treatments and SARS‐CoV‐2 vaccination on brain atrophy after SARS‐CoV‐2 infection. In a subgroup analysis, we excluded six MS patients who had received anti‐SARS‐CoV‐2 treatments (4/6) and/or had been vaccinated against SARS‐CoV‐2 before infection (4/6). Eight unvaccinated and anti‐SARS‐CoV‐2 untreated patients with 58 MRI scans remained in whom we observed more pronounced atrophy in the parahippocampal gyri (*p* = 0.037, Figure 2), compared with the small subgroup of six MS patients who had previously received a vaccination and/or anti‐SARS‐CoV‐2 treatment (*p* = 0.46, Figure 3).

## 4 CONCLUSION AND DISCUSSION

We analyzed brain atrophy rates in patients with MS after infection with SARS‐CoV‐2. A patient‐specific baseline was derived from longitudinal preinfection MRIs and compared with postinfection measures. No evidence for an increased global atrophy rate was observed, but regional atrophy in the parahippocampal gyri was increased, confirming the main finding of the UK Biobank study.^1^ Our proposed approach allows adjustment for the intraindividual brain atrophy rate prior to SARS‐CoV‐2 infection and shows whether the brain atrophy accelerates during or after SARS‐CoV‐2 infection. Such an analysis can complement results from studies like those presented by Douaud et al.,^1^ where a single MRI scan before and after SARS‐CoV‐2 infection was available for a large number of subjects but offered no possibility to adjust for the intraindividual and regional atrophy rate. We call for further studies to reproduce these investigations in MS registries worldwide. We also encourage the pooling of the results across institutes to enable unmatched investigations of SARS‐CoV‐2 effects and potential effects of anti‐SARS‐CoV‐2 vaccinations and anti‐SARS‐CoV‐2 treatments on brain atrophy. Such continuous collaborative effort of the MS community could fill important missing points in the knowledge of SARS‐CoV‐2 itself as well as the interplay between SARS‐CoV‐2 and MS.^14^

Scripts for the analysis are publicly available (https://github.com/SCAN‐NRAD/BrainAtrophyRate), and the software for atrophy analysis can be downloaded from https://github.com/SCAN‐NRAD/DL‐DiReCT; we welcome direct contact via the corresponding author.

## AUTHOR CONTRIBUTIONS

CF, MR, PR, CR, AS, and RH designed the study. CF, AS, RH, and PR identified the patients and provided clinical information. MR wrote code and analyzed the imaging and clinical data. CF, RH, AS, MR, PR, and CR drafted the manuscript. LD, RW, and AC critically revised the manuscript. All authors have critically revised the final version of the manuscript.

## CONFLICT OF INTEREST

**RW, MR, PR, and CR** have nothing to disclose. **CF** received speaker honoraria and/or travel compensation for activities with Biogen, Sanofi Genzyme, Novartis, and Merck and research support from Chiesi, not related to this work. He reports no conflict of interest related to this manuscript. **LD** has received travel grants from Bayer, Biogen, Roche, and Merck as well as speaker honoraria from Merck and Biogen, not related to this work. **AC** has served on advisory boards for and received funding for travel or speaker honoraria from Actelion‐Janssen, Almirall, Bayer, Biogen, Celgene, Sanofi Genzyme, Merck, Novartis, Roche, and Teva, all for hospital research funds; and research support from Biogen, Genzyme, and UCB. Chan A is an associate editor of the *European Journal of Neurology* and serves on the editorial board for *Clinical and Translational Neuroscience* and as a topic editor for the *Journal of International Medical Research*. **AS** received speaker honoraria and/or travel compensation for activities with Bristol Myers Squibb, CSL Behring, Novartis, Roche, and research support by Baasch Medicus Foundation, the Medical Faculty of the University of Bern, and the Swiss MS Society. She serves on the Editorial Board of *Frontiers in Neurology—Multiple Sclerosis and Neuroimmunology*. All are not related to this work. **RH** received speaker/advisor honoraria from Merck, Novartis, Roche, Biogen, Alexion, Sanofi, Janssen, Bristol‐Myers Squibb, and Almirall. He has received research support within the last 5 years from Roche, Merck, Sanofi, Biogen, Chiesi, and Bristol‐Myers Squibb. He has also received research grants from the Swiss MS Society. He also serves as an associate editor for the *Journal of Central Nervous System Disease*. None of these are related to this work.

## Supporting information


Figure S1
Figure S2
Figure S3
Figure S4

## Data Availability

The authors take full responsibility for the data, the analyses and interpretation, and the conduct of the research; and have full access to all of the data. The authors have the right to pub‐lish any and all data, separate and apart from the guidance of any sponsor. Data and material are available upon reasonable request via the corresponding author.
