# Supplementary material for: Multiple sclerosis as a model to investigate SARS‐CoV‐2 effect on brain atrophy
Source: CNS Neurosci Ther. 2022 Dec 7;29(2):538–43. doi: 10.1111/cns.14050 (PMC9873510; doi:10.1111/cns.14050)
Supplement: Supplementary file 1 — Figure S1 Figure S2 Figure S3 Figure S4 [file CNS-29-538-s001.docx]

**Supplementary information**


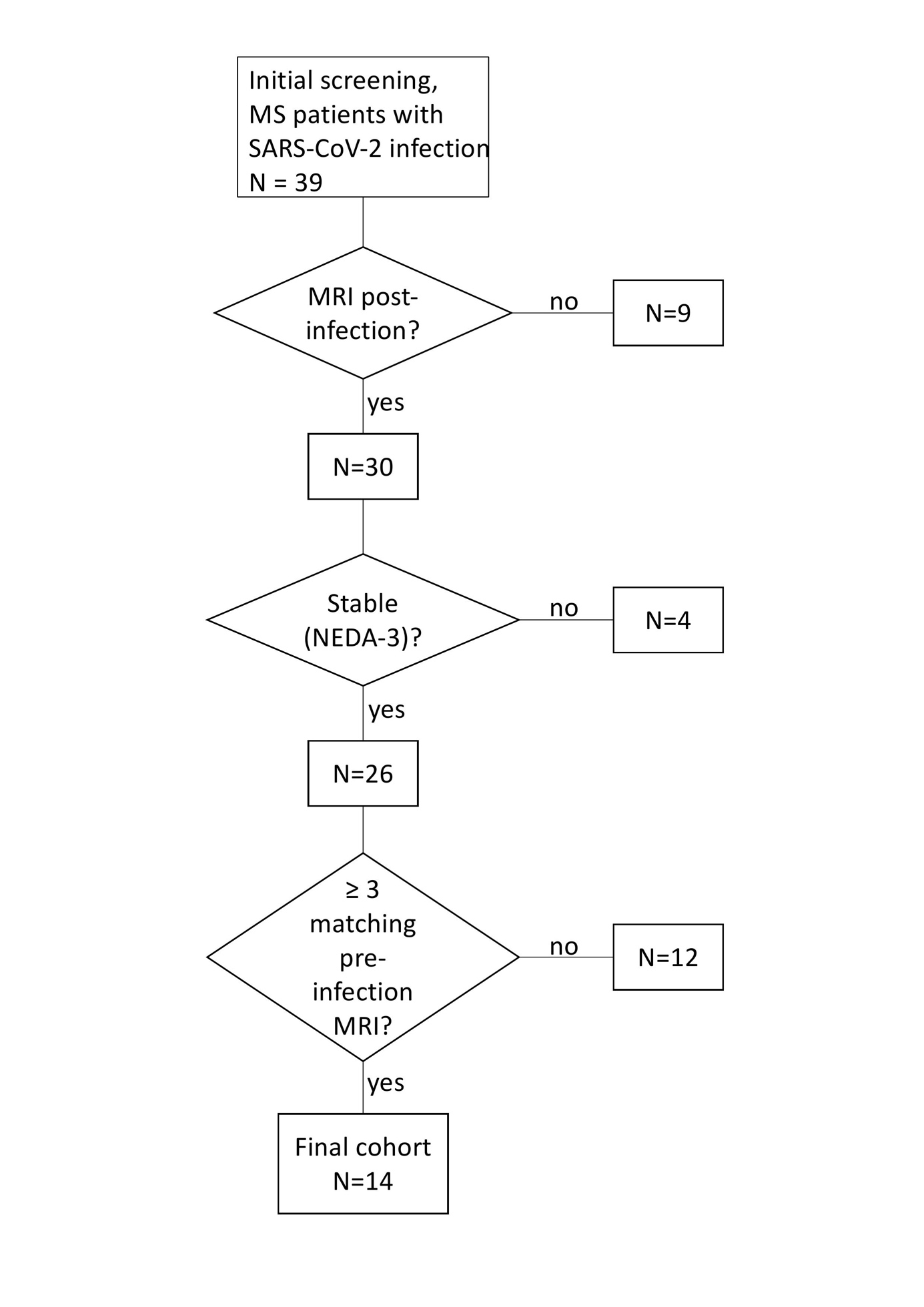


Figure S1: Flowchart depicting inclusion of patients in the cohort.

*
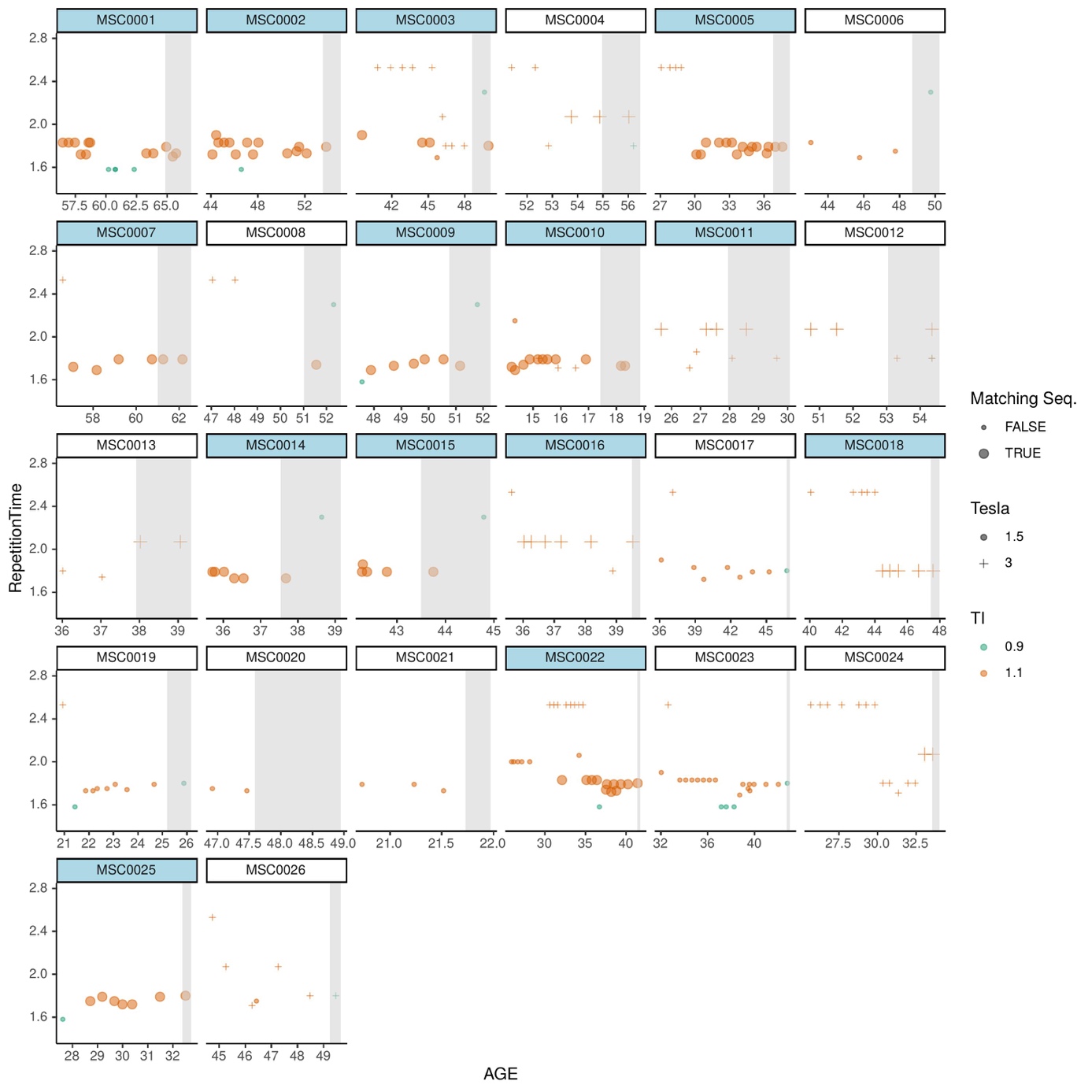
*

*Figure S2:* Initial screening of MRI scans to identify those with matching MR sequence parameters to be included in the analysis. Inversion time (TI) is colour-coded and repetition time is plotted on the y-axis. Matching time points are depicted with larger symbols. Patients included in the final analysis are highlighted in light blue. Grey-shaded areas represent post-SARS-CoV-2 time points.


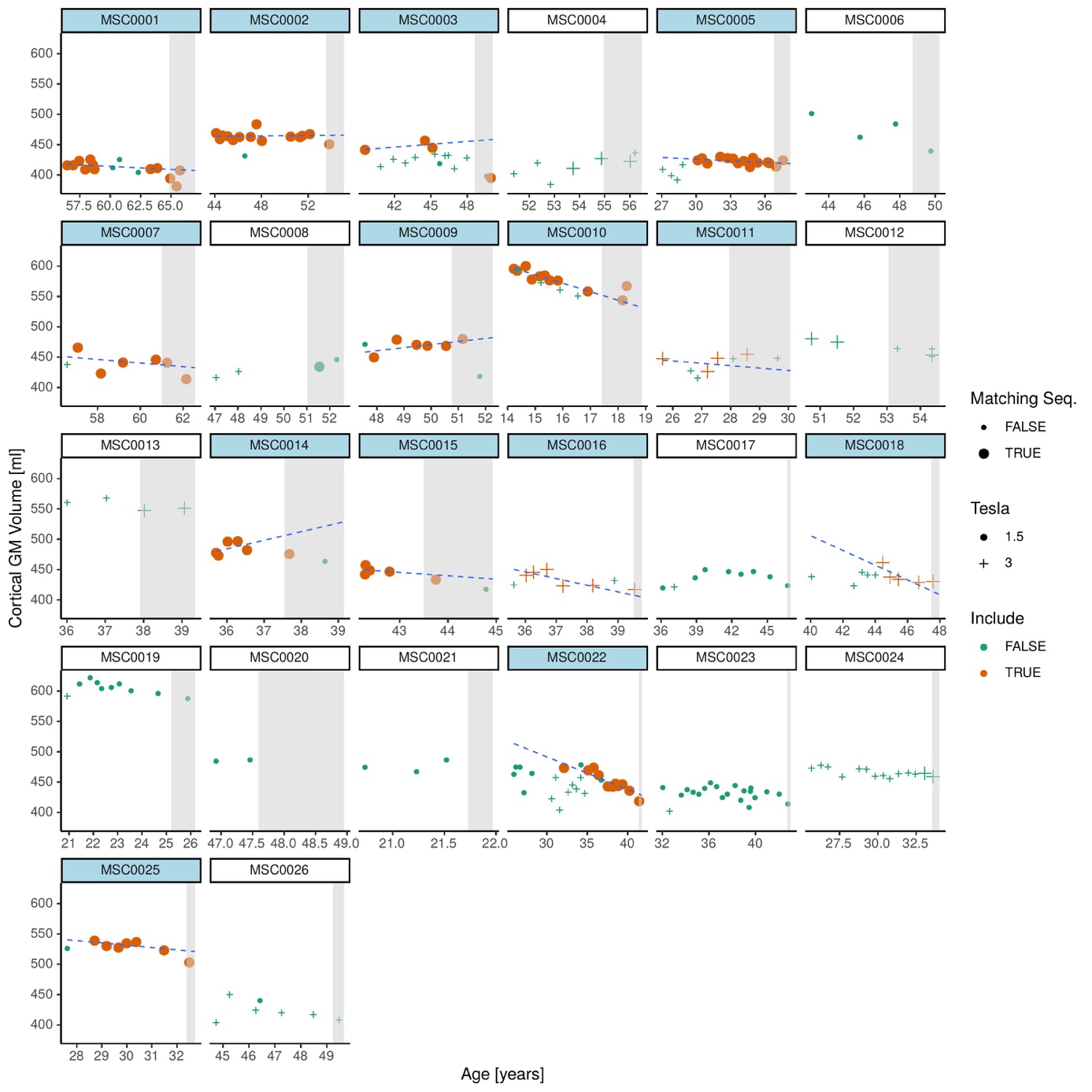


Figure S3: Individual results for the cortical grey matter volume for all patients. Regression lines were fitted to matching MRI scans (in red). Patients were included if at least three eligible pre-SARS-CoV-2 images were available. Patients included in the final analysis are highlighted in light blue. Grey-shaded areas represent post-SARS-CoV-2 time points.


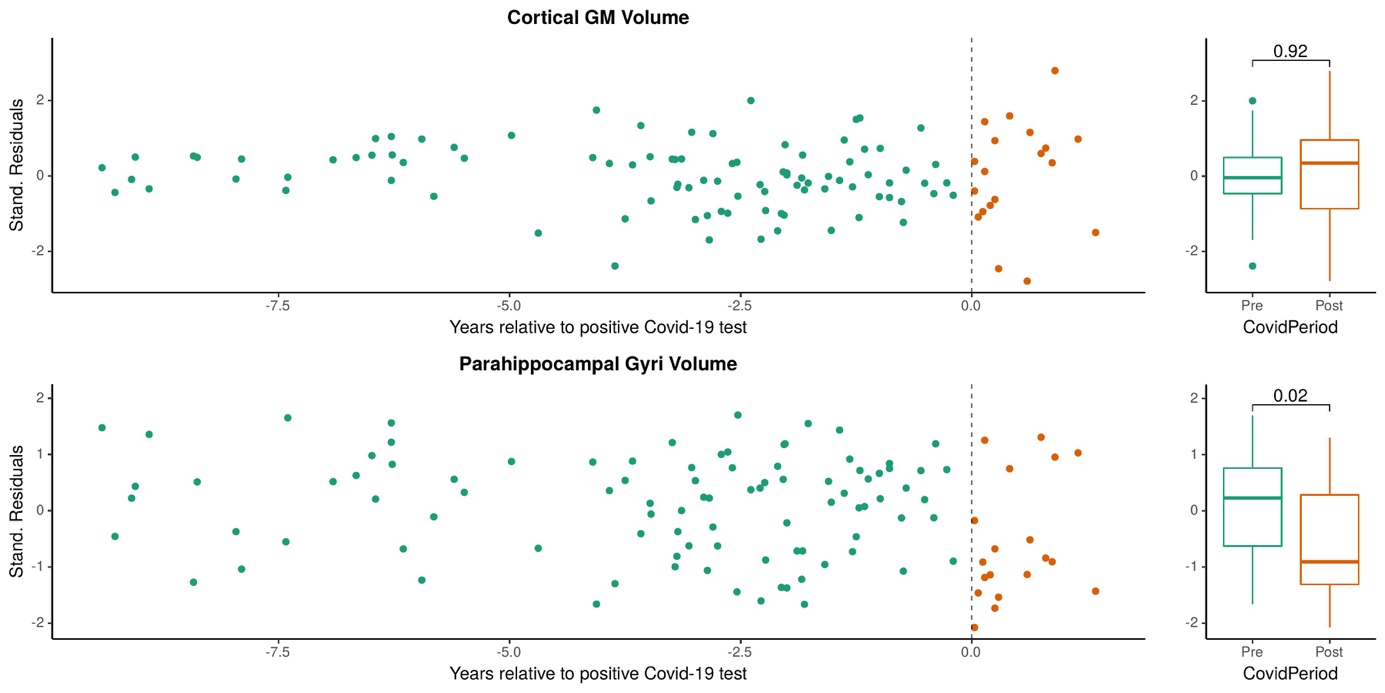


Figure S4: Results using DL+DiReCT as an alternative analysis tool. This figure corresponds to Figure 1 of the main text, where results are shown for FreeSurfer volumes.

Abbreviations

*GM*: grey matter

*Covid-19*: coronavirus disease 2019

*MRI*: magnetic resonance imaging

*MS*: Multiple sclerosis

*N*: number of observations

*NEDA-3*: No evidence of disease activity-3

*SARS-CoV-2*: severe acute respiratory syndrome coronavirus type 2

*Seq.*: Sequence

*Stand.*: standardized

*TI*: inversion time
